# Iatrogenic Aortic Stenosis During a Case of Aortic Paravalvular Leak Closure

**DOI:** 10.1016/j.shj.2022.100022

**Published:** 2022-03-25

**Authors:** Jennifer Jdaidani, Dounia Z. Iskandarani, Omar Chaabo, Pierre M. Sfeir, Ziyad M.B. Ghazzal, Walid Gharzeddine, Fadi J. Sawaya

**Affiliations:** a Division of Cardiology, American University of Beirut Medical Center, Beirut, Lebanon; b Division of Cardiothoracic Surgery, American University of Beirut Medical Center, Beirut, Lebanon

**Keywords:** Aortic paravalvular leak closure, Aortic stenosis, Iatrogenic aortic stenosis, Paravalvular leak, Percutaneous device closure of paravalvular leak

## Case Presentation

We report the case of a 70-year-old male patient with prior aortic valve replacement (AVR) using a Carpentier-Edwards PERIMOUNT Magna Ease pericardial bioprosthesis 19 mm valve for severe aortic stenosis. He presented to our institution 2 ​years later with severe hemolytic anemia requiring regular blood transfusions and pulmonary edema hospitalizations.

Transthoracic echocardiography showed a stenotic valve with a mean pressure gradient of 37 mm Hg (postoperative gradient of 28 mm Hg), as well as a torrential central and paravalvular aortic regurgitation (Supplemental Video 1). Given the small valve implanted and the high probability of recurrent patient-prosthesis mismatch, the heart team opted for a strategy of percutaneous paravalvular leak (PVL) closure to address the hemolytic anemia and aortic PVL, with a staged valve-in-valve transcatheter AVR with bioprosthetic valve fracture, to achieve the biggest effective orifice area.

Under general anesthesia and transesophageal echocardiography guidance, multiple PVL leaks were crossed using MP/AL1 and Terumo wires, leading to the insertion of 3 large 13 × 5 mm Amplatzer Vascular Plugs III (AVP III; Supplemental Videos 2-5). The paravalvular regurgitation significantly decreased from torrential to mild with a small residual anterior jet (Supplemental Videos 6 and 7).

AVP III has an oblong cross-sectional shape made of multiple Nitinol mesh layers and has extended rims. Device rims extending beyond the device body enable covering a larger surface area, making AVP III our device of choice in big crescentic and extensive leaks like this case.

**Figure 1:**
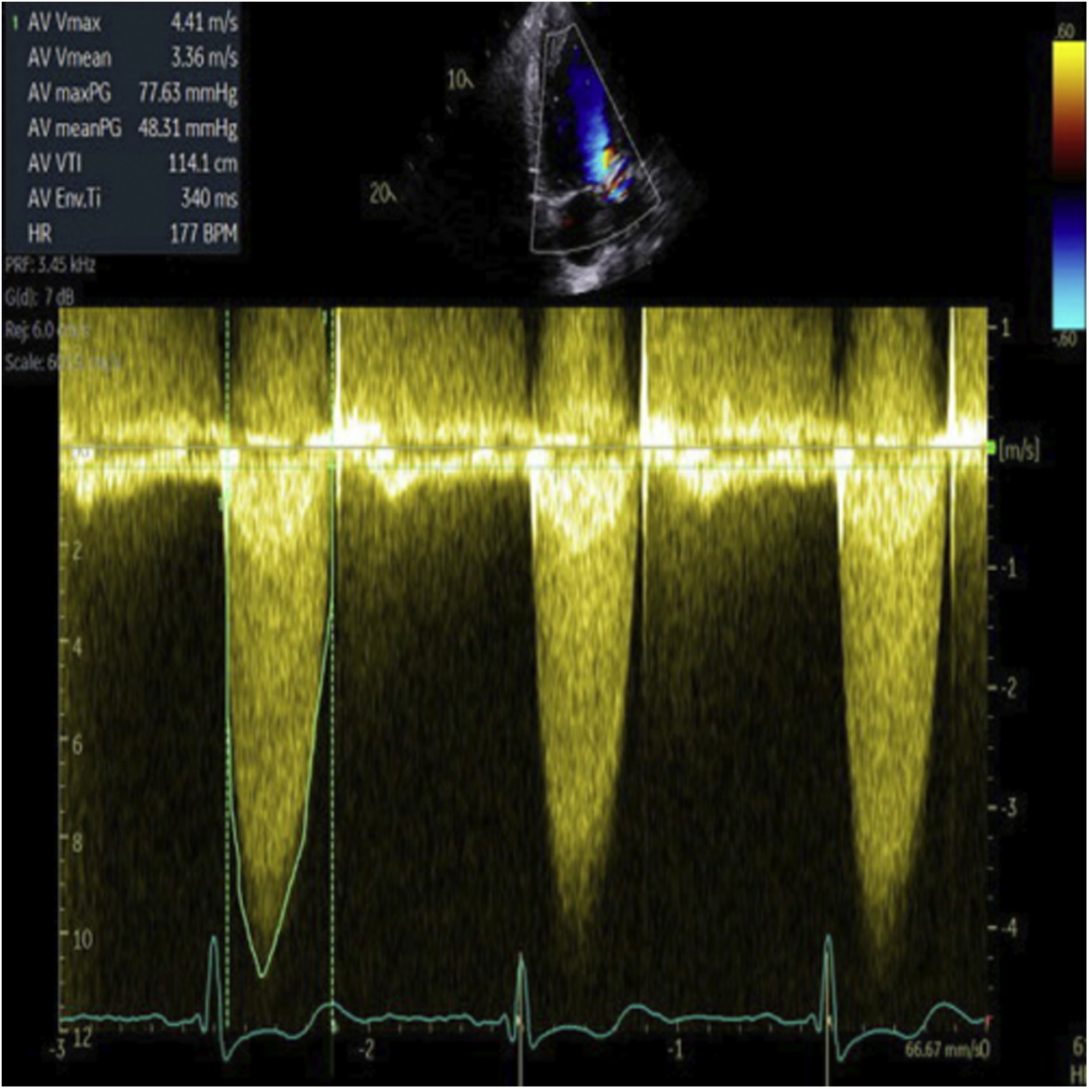
**Severe aortic stenosis on transthoracic echocardiography: Aortic bioprosthetic stenosis with mean pressure gradient of 48 mm Hg**.

Despite a mild hemolytic anemia improvement (lactate dehydrogenase from 1150 to 678 IU), the patient was readmitted for pulmonary edema 2 ​months later. Transthoracic echocardiography showed mild central aortic regurgitation and mild PVL, however, with an increase in transaortic mean pressure gradient to 48 mm Hg and a restricted movement of one of the cusps (Figure 1).

Given the persistent elevated lactate dehydrogenase with some blood transfusion requirements, we believed a surgical AVR would be the best approach at this point.

**Figure 2:**
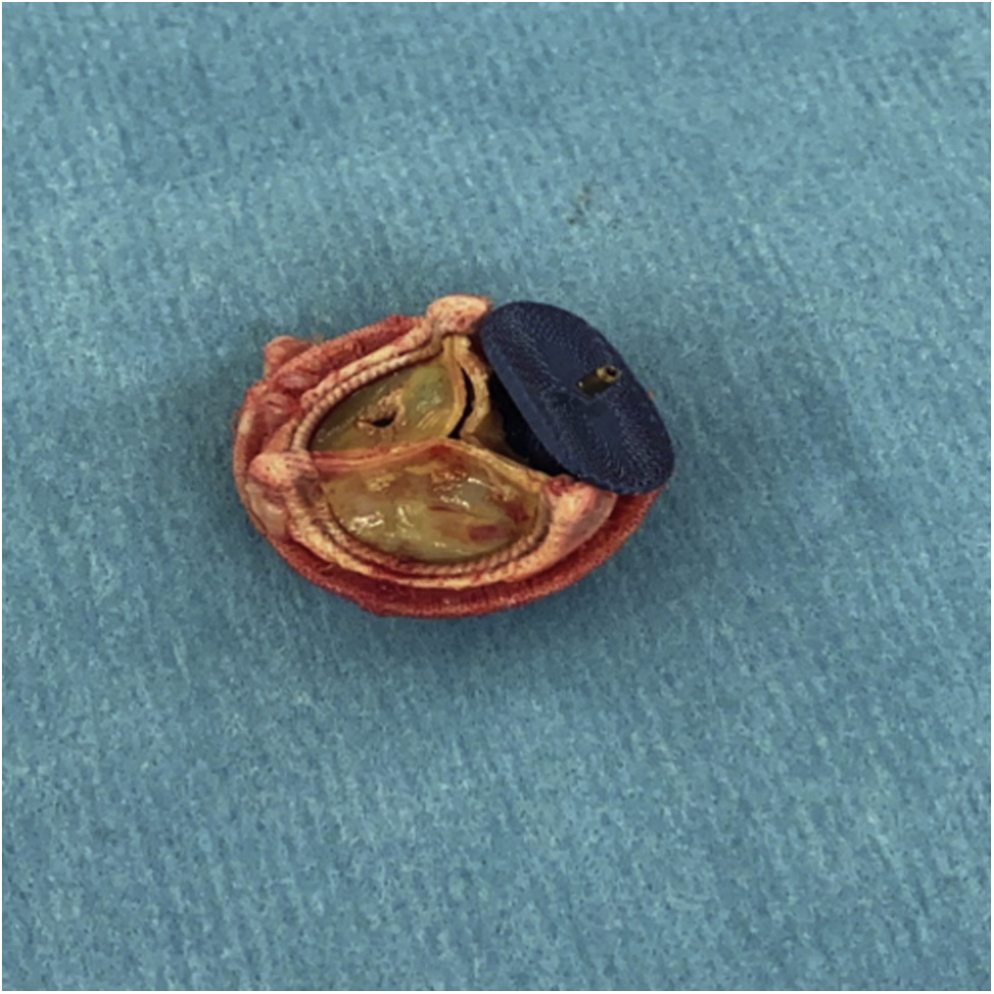
**Intravalvular Plug: Amplatzer Vascular Plug III (AVP III) plug sealing an aortic leaflet perforation.** Adjacent leaflet perforation in another cusp is appreciated as well.

Intra-operatively, we found 2 AVP III plugs well seated in the perivalvular space with, to our surprise, the third plug located in an intravalvular position obstructing flow while sealing a perforated leaflet (Figure 2 and Supplemental Video 8). Adjacent leaflet perforation was also appreciated in another cusp. The valve was replaced with an appropriate size 23 mm Carpentier-Edwards PERIMOUNT Magna Ease pericardial bioprosthesis.

This case was worthwhile reporting, as we failed to perceive the leaflet perforation and the intravalvular location of one of the plugs with transesophageal echocardiography, although appreciated retrospectively when the plug was detected moving with the leaflet in systole and diastole (Supplemental Videos 9 and 10). Proper imaging and careful panning from left anterior oblique to right anterior oblique in this small 19 mm valve could have appreciated the location of the crossing wire and avoided this iatrogenic aortic stenosis. Moreover, transgastric images would have allowed us to appreciate the increase in aortic gradients.

## Consent Statement

Patient consent for publication has been obtained by the authors.

## Funding

The authors have no funding to report.

## Disclosure statement

F.J.S. is a TAVR proctor for Edwards Lifesciences, Medtronic, Abbot Vascular, and Boston Scientific. All remaining authors have nothing relevant to disclose.

## Supplementary Material

Supplemental Video 1 Baseline transthoracic echocardiography: Torrential central and paravalvular aortic regurgitation.

Supplemental Video 2 Fluoroscopic angiography: Aortic angiogram showing torrential aortic regurgitation.

Supplemental Video 3 Fluoroscopic first plug insertion: 13 × 5 mm AVP III deployment.

Supplemental Video 4 Fluoroscopic second plug insertion: 13 × 5 mm AVP III deployment.

Supplemental Video 5 Fluoroscopic third plug insertion: 13 × 5 mm AVP III deployment.

Supplemental Video 6 Intraprocedural transesophageal echocardiography: Significant improvement of the aortic regurgitation from torrential to mild.

Supplemental Video 7 Aortic regurgitation before and after paravalvular leak closure: Significant improvement of the paravalvular regurgitation from torrential to mild after PVL closure.

Supplemental Video 8 Intravalvular location plug with a second leaflet perforation: AVP III plug sealing an aortic leaflet perforation. Another leaflet perforation in another cusp is appreciated as well.

Supplemental Video 9 Retrospective fluoroscopy: Plug motion with valve opening and closing in systole and diastole.

Supplemental Video 10 Retrospective transthoracic echocardiography: Central location of one of the plugs in the right coronary cusp.

